# Knockout of Murine *Mamld1* Impairs Testicular Growth and Daily Sperm Production but Permits Normal Postnatal Androgen Production and Fertility

**DOI:** 10.3390/ijms18061300

**Published:** 2017-06-19

**Authors:** Mami Miyado, Kaoru Yoshida, Kenji Miyado, Momori Katsumi, Kazuki Saito, Shigeru Nakamura, Tsutomu Ogata, Maki Fukami

**Affiliations:** 1 Department of Molecular Endocrinology, National Research Institute for Child Health and Development, Tokyo 157-8535, Japan; miyado-m@ncchd.go.jp (M.M.); katsumi-m@ncchd.go.jp (M.K.); saito-kz@ncchd.go.jp (K.S.); nakamura-sh@ncchd.go.jp (S.N.); tomogata@hama-med.ac.jp (T.O.); 2 Faculty of Biomedical Engineering, Toin University of Yokohama, Yokohama 225-8502, Japan; yoshidak@toin.ac.jp; 3 Department of Reproductive Biology, National Research Institute for Child Health and Development, Tokyo 157-8535, Japan; miyado-k@ncchd.go.jp; 4 Department of NCCHD Child Health and Development, Graduate School, Tokyo Medical and Dental University, Tokyo 113-8510, Japan; 5 Department of Comprehensive Reproductive Medicine, Graduate School, Tokyo Medical and Dental University, Tokyo 113-8510, Japan; 6 Department of Pediatric Urology, Jichi Medical University, Children’s Medical Center Tochigi, Tochigi 329-0498, Japan; 7 Department of Pediatrics, Hamamatsu University School of Medicine, Hamamatsu 431-3192, Japan

**Keywords:** androgen, knockout mouse, mutation, reproduction, testis

## Abstract

MAMLD1 has been implicated in testicular function in both human and mouse fetuses. Although three patients with *MAMLD1* mutations were reported to have hypergonadotropic hypogonadism in their teens, the functional significance of MAMLD1 in the postnatal testis remains unclear. Here, we analyzed the phenotype of *Mamld1* knockout (KO) male mice at reproductive ages. The reproductive organs of KO male mice were morphologically unremarkable, except for relatively small testes. Seminiferous tubule size and number of proliferating spermatogonia/spermatocytes were reduced in the KO testis. Daily sperm production of KO mice was mildly attenuated, whereas total sperm counts in epididymal semen remained normal. Sperm motility and morphology, as well as androgen levels in serum and testicular tissues and the number of pups born from cross-mated wildtype (WT) female mice, were comparable between WT and KO male mice. These results indicate that MAMLD1 contributes to the maintenance of postnatal testicular growth and daily sperm production but is dispensable for androgen biosynthesis and fertility. MAMLD1 likely plays supporting roles in multiple and continuous steps of male reproduction.

## 1. Introduction

*MAMLD1* on the human X chromosome is a causative gene for hypospadias [1]. *MAMLD1* mutations, which are presumed to affect androgen biosynthesis in the fetal testis, result in hypospadias. Indeed, in vitro knockdown of *Mamld1* significantly reduced testosterone production in murine Leydig tumor cells [2]. Furthermore, genetic knockout (KO) of murine *Mamld1* decreased the expression of several Leydig cell-specific genes in the fetal testis [3]. Although *Mamld1* KO mice exhibited no hypospadias, phenotypic differences between human patients and KO mice can be explained by inter-species differences in steroid metabolism [3].

To date, pathogenic mutations of *MAMLD1* have been identified primarily in infants or prepubertal boys with hypospadias [1,4,5,6,7]. It remains unknown whether MAMLD1 is indispensable for testicular function at later ages. Recently, Fujisawa et al. performed a long-term follow-up study of three patients with hypospadias due to *MAMLD1* nonsense mutations [8]. Although these patients manifested no hormonal abnormalities during infancy, they showed mild hypergonadotropic hypogonadism in their teens [8]. In addition, physical examinations at 7–18 years of age revealed relatively small testes and micropenis [8]. Two of the three patients presented with testicular microlithiasis [8], an ultrasound finding often associated with testicular dysfunction [9]. These results suggest that MAMLD1 is involved in postnatal testicular function. Consistent with this, we detected a clear *Mamld1* expression in testes of postnatal mice [3]. However, the abovementioned notion is based on data from only three patients and therefore needs to be validated in further studies. In this regard, although previous studies have shown that *Mamld1* KO male mice are fertile [3,10], detailed testicular function has yet to be studied in these adult mice. The aim of this study was to clarify the phenotype of *Mamld1* KO male mice at reproductive ages.

## 2. Results

### 2.1. Anatomical, Histological, and Immunohistochemical Examinations of Reproductive Organs of Mamld1 Knockout (KO) Mice

**Figure 1:**
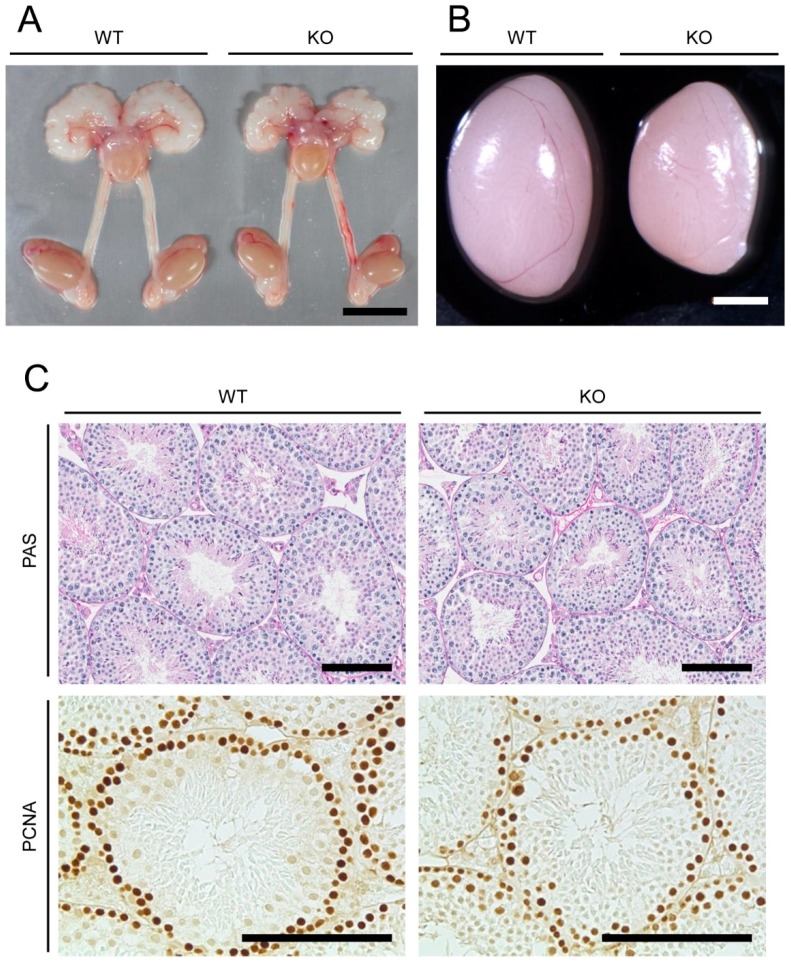
Morphological analyses. (**A**) Anatomy of male reproductive organs of wildtype (WT) and *Mamld1* knockout (KO) mice at 20 weeks of age. Scale bars = 1 cm; (**B**) Testis morphology of WT and KO mice at 20 weeks of age. Scale bars = 2 mm; (**C**) Testis sections stained with periodic acid-Schiff (PAS) and proliferating cell nuclear antigen (PCNA) antibody in WT and KO mice at 8 weeks of age. Scale bars = 100 μm.

We analyzed reproductive phenotypes of *Mamld1* KO male mice generated in our previous study [3]. The KO mice and their wildtype (WT) littermates at 5–20 weeks of age were studied. As reported previously [3], *Mamld1* KO male mice were viable and exhibited no hypospadias. The reproductive organs of KO mice were morphologically unremarkable, except for relatively small testes (Table 1 and Figure 1A,B). Weights of the epididymis and other reproductive organs were comparable between WT and KO animals (Table 1).

The testes of *Mamld1* KO mice showed no gross histological abnormalities (Figure 1C). Spermatogonia, primary spermatocytes, round spermatids, elongated spermatids, and Sertoli cells were observed in the seminiferous tubules of both WT and KO testes. However, the average short-axis diameter of the seminiferous tubules was lower in KO mice than in WT littermates (169.0 ± 1.9 μm vs. 188.9 ± 3.2 μm; *p* = 0.0060). In addition, cells expressing proliferating cell nuclear antigen (PCNA), a marker for proliferating spermatogonia and spermatocytes [11,12], were less frequently observed in the seminiferous tubules of KO mice than in those of WT animals (Figure 1C).

### 2.2. Sperm Analysis of Mamld1 KO Mice

Daily sperm production was estimated based on the number of spermatids per testis [13]. Daily sperm production in KO mice at 8 weeks of age accounted for 70–80% of that in WT animals, whereas total sperm counts in semen samples obtained from the epididymis were comparable between WT and KO animals (Table 2).

**Figure 2:**
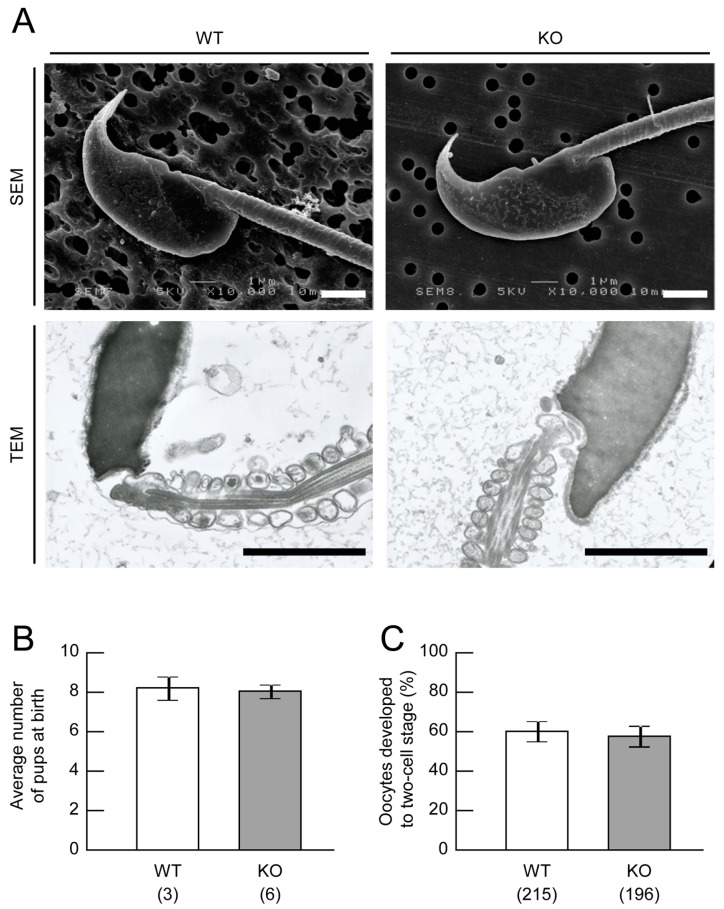
Sperm analyses. (**A**) Sperm morphology observed by scanning electron microscopy (SEM) and transmission electron microscopy (TEM). WT, wildtype; KO, knockout. Scale bars = 2 μm; (**B**) Average number of pups born from WT mothers. The numbers in parentheses indicate the numbers of male mice examined. Values are the mean ± standard error of the mean; (**C**) The percentage of oocytes developed to two-cell stage 24 hours after in vitro fertilization. The numbers in parentheses indicate the numbers of WT oocytes examined. Values are the mean ± standard error of the mean.

Next, we examined the quality of sperm collected from the epididymides of WT and KO mice. Sperm motility of mice at 8 weeks of age was assessed using a computer-assisted system [14] and sperm morphology of mice at 10 weeks of age was observed by scanning and transmission electron microscopy. As shown in Table 3 and Figure 2A, sperm motility and morphology in KO mice were similar to those observed in WT mice.

### 2.3. Androgen Measurement of Mamld1 KO Mice

We measured concentrations of androgens in serum and testicular samples of WT and KO mice at 8 weeks of age. Steroid levels were quantified by liquid chromatography-tandem mass spectrometry. As shown in Table 4, serum levels of androstenedione, testosterone, and dihydrotestosterone were comparable between WT and KO mice. Likewise, there were no significant differences in intra-testicular testosterone values between WT and KO mice.

### 2.4. Fertility Assessment of Mamld1 KO Mice

We investigated the fertility of *Mamld1* KO male mice. First, we cross-mated WT female mice to WT and KO male mice aged 8–13 weeks. This experiment resulted in the birth of a comparable number of pups from WT and KO male mice (Figure 2B). Then, we examined the in vitro fertilization abilities of sperm collected from the epididymides of WT and KO male mice at 10–11 weeks of age. The results indicated that KO sperm retains normal fertilization abilities (Figure 2C).

## 3. Discussion

The present study demonstrated that *Mamld1* deficiency produces small testes in mice at reproductive ages. Moreover, the number of PCNA-positive cells in the seminiferous tubules and daily sperm production was reduced in *Mamld1* KO mice. These findings imply that MAMLD1 is involved in germ cell proliferation in postnatal mice. Notably, however, epididymal sperm concentration and fertility remained normal in KO mice. Furthermore, testosterone levels in the blood and testis were comparable between WT and KO mice. Collectively, our data suggest that in postnatal mice, MAMLD1 enhances testicular growth and spermatogenesis, although it is not essential for androgen production and fertility. In this regard, we have previously shown that *Mamld1* deficiency during the fetal period significantly reduces the mRNA expression of multiple genes in fetal Leydig cells, but permits masculinization of external genitalia [3]. Although the present study showed no significant difference in the androgen levels between WT and KO adult mice, this may be due to inter-individual variability or the small number of biological replicates. Further studies are necessary to clarify whether the expression levels of steroidogenic enzyme genes such as *Star* and *Cyp11a1* are altered in postnatal KO testis. Collectively, it appears that MAMLD1 is involved in the testicular function during fetal period and at reproductive ages.

The results of this study are consistent with those of a previous study of three human patients with *MAMLD1* mutations. Fujisawa et al. documented relatively small testes and testicular microlithiasis in pubertal boys with *MAMLD1* nonsense mutations [8]. However, unlike *Mamld1* KO mice, these human patients invariably exhibited subnormal androgen production during puberty. This suggests that MAMLD1 has species-specific roles in the testis. Phenotypic differences between human patients and KO mice may reflect inter-species differences in steroid metabolism, as suggested previously [3]. Furthermore, because the patients reported by Fujisawa et al. manifested age-dependent deterioration of testicular function [8], it is possible that *Mamld1* KO mice develop progressive testicular dysfunction at later ages.

In conclusion, the results of this study indicate that murine MAMLD1 contributes to the maintenance of postnatal testicular growth and daily sperm production, but is dispensable for androgen biosynthesis and fertility. These results, together with those of previous studies [1,3,8], imply that MAMLD1 plays supporting roles in multiple steps of male reproduction in both humans and mice.

## 4. Materials and Methods

### 4.1. Animal Care

This study was approved by the Animal Ethics Committee of National Research Institute for Child Health and Development (project number: A2008-001; 1 April 2008). All experiments were performed in accordance with the institutional guidelines of the care and use of laboratory animals. All mice were housed under specific pathogen-free controlled conditions with a 12 h light-dark cycle. Food and water were available ad libitum.

### 4.2. Morphological, Histological, and Immunohistochemical Examinations of Reproductive Organs of Mamld1 KO Mice

Mice lacking *Mamld1* were generated in our previous study [3]. In these mice, exon 3 of *Mamld1* was replaced by a neo-cassette. The mice were backcrossed with the C57BL/6N strain (Sankyo Labo Service Corp. Inc., Tokyo, Japan). In the present study, we analyzed the phenotypes of KO male mice at 5–20 weeks of age. As controls, age-matched WT littermates were analyzed.

Reproductive organs of mice at 5, 8, and 20 weeks of age were weighed. The right testes of 8-week-old mice were fixed with the Bouin’s solution (Mutoh Chemical, Tokyo, Japan) or 4% paraformaldehyde (PFA), dehydrated, embedded in paraffin, and sectioned. Bouin’s-fixed samples (3-μm thick slices) were stained with periodic acid-Schiff. The short-axis diameters of 20 randomly selected seminiferous tubule sections were measured for each animal (*n* = 3). We also stained PFA-fixed samples (6-μm thick slices) with an anti-PCNA antibody (PC10 clone, diluted 1:200; Dako, Copenhagen, Denmark).

### 4.3. Sperm Analysis of Mamld1 KO Mice

Right testes of WT and KO mice at 8 weeks of age were isolated, placed in liquid nitrogen, and kept at −80 °C. We removed the tunica albuginea from the testis and homogenized the testis. Daily sperm production was estimated based on the number of spermatids per testis [13]. We counted spermatids at steps 14–16 using a hemocytometer.

To examine sperm quality, we collected samples from the epididymides of WT and KO mice at 8 and 10 weeks of age. Sperm motility was assessed using a computer-assisted system (Hamilton Thorne, Inc., Beverly, MA, USA), as described previously [14]. Sperm morphology was observed using a scanning electron microscope and a transmission electron microscope (Hanaichi UltraStructure Research Institute, Okazaki, Japan).

### 4.4. Androgen Measurement of Mamld1 KO Mice

We measured the concentrations of androstenedione, testosterone, and dihydrotestosterone in serum and testicular samples of WT and KO mice at 8 weeks of age. Blood samples were drawn from the right ventricles of the hearts of euthanized mice and centrifuged. Each testis was isolated, weighed, and frozen in liquid nitrogen. Samples were stored at −80 °C until steroid measurement. Steroid levels were quantified by liquid chromatography-tandem mass spectrometry (ASKA Pharma Medical, Kanagawa, Japan).

### 4.5. Fertility Assessment of Mamld1 KO Mice

We investigated fertility of *Mamld1* KO male mice. To assess in vivo fertilization ability, we cross-mated WT and KO male mice at 8–13 weeks of age with WT C57BL/6N female mice (8–10 weeks of age). The number of pups was counted on the day of delivery.

To assess in vitro fertilization ability, we collected sperm from the epididymis of WT and KO male mice at 10–11 weeks of age and incubated the samples with oocytes collected from WT C57BL/6N female mice (8 weeks of age). After 24 h culture, the percentage of oocytes developed to two-cell stage was calculated, as described previously [15].

### 4.6. Statistical Analysis

Statistical differences in mean values between two groups were examined by Student’s *t*-test or Mann-Whitney’s *U*-test. *p* values less than 0.05 were considered significant. Data are expressed as the mean ± standard error of the mean.

## Figures and Tables

**Table 1:** Weight of reproductive organs of mice at 5, 8 and 20 weeks of age.

Organs	Genotype		Statistical Significance (*p* Values)
	WT	*Mamld1* KO	
At 5 weeks of age (*n*)	6	6	
Body (g)	21.0 ± 0.6	21.4 ± 0.3	0.75
Testes (mg)	134.5 ± 3.6	120.7 ± 1.3	0.0046
Epididymides (mg)	35.7 ± 1.6	35.7 ± 0.9	0.99
At 8 weeks of age (*n*)	9	8	
Body (g)	22.6 ± 0.4	25.2 ± 0.5	0.0015
Testes (mg)	169.8 ± 4.6	151.0 ± 2.7	0.0038
Epididymides (mg)	54.7 ± 1.3	55.2 ± 0.9	0.79
At 20 weeks of age (*n*)	6	7	
Body (g)	30.2 ± 0.6	34.8 ± 1.4	0.017
Testes (mg)	208.7 ± 7.6	187.2 ± 7.3	0.033
Epididymides (mg)	88.9 ± 2.9	86.7 ± 1.0	0.48
Coagulating glands (mg)	29.4 ± 1.4	30.2 ± 1.2	0.68
Prostate glands (mg)	39.6 ± 2.8	41.2 ± 2.3	0.67
Seminal vesicles (mg)	304.0 ± 11.5	320.5 ± 5.9	0.21
Preputial glands (mg)	108.0 ± 4.5	95.7 ± 10.6	0.34

WT, wildtype; KO, knockout. The results are expressed as the mean ± standard error of the mean.

**Table 2:** Sperm analysis of mice at 8 weeks of age.

Parameters	WT	*Mamld1* KO	Statistical Significance (*p* Values)
Daily sperm production per testis (×10^6^)	6.45 ± 0.24 (*n* = 6)	4.67 ± 0.24 (*n* = 8)	0.00026
Daily sperm production per gram of testis (×10^7^)	7.85 ± 0.42 (*n* = 6)	6.48 ± 0.24 (*n* = 8)	0.011
Epididymal sperm count (×10^7^/mL)	1.05 ± 0.06 (*n* = 7)	1.04 ± 0.08 (*n* = 6)	0.89

WT, wildtype; KO, knockout. The results are expressed as the mean ± standard error of the mean.

**Table 3:** Qualitative assessment of sperm of mice at 8 weeks of age.

Parameters	WT (*n* = 4)	*Mamld1* KO (*n* = 3)	Statistical Significance (*p* Values)
Total motility (%)	97.7 ± 0.3	94.3 ± 0.9	0.94
Progressive motility (%)	43.0 ± 5.4	49.7 ± 2.8	0.28
Rapid motility (%)	55.0 ± 5.1	57.7 ± 3.2	0.21
Static cell (%)	2.3 ± 0.3	4.0 ± 1.2	0.46
Average path velocity (mm/s)	127.6 ± 2.7	133.1 ± 2.3	0.37
Amplitude of lateral head displacement (mm)	8.3 ± 0.02	8.6 ± 0.3	0.17
Hyperactivation (%)	16.6 ± 1.7	23.0 ± 2.4	0.071

WT, wildtype; KO, knockout. The results are expressed as the mean ± standard error of the mean. At least 200 sperm were examined for each mouse.

**Table 4:** Steroid hormone levels of mice at 8 weeks of age.

Steroids	WT	*Mamld1* KO	Statistical Significance (*p* Values)
Serum androstenedione (pg/mL)	97.15 ± 41.67 (*n* = 8)	243.80 ± 74.26 (*n* = 10)	0.25
Serum testosterone (ng/mL)	2.06 ± 0.95 (*n* = 10)	4.47 ± 1.69 (*n* = 10)	0.50
Serum dihydrotestosterone (pg/mL)	105.30 ± 34.46 (*n* = 10)	158.90 ± 51.58 (*n* = 10)	0.50
Intra-testicular testosterone (ng/testis)	15.21 ± 5.66 (*n* = 3)	30.34 ± 8.75 (*n* = 3)	0.22
Intra-testicular testosterone (ng/mg of testis)	0.22 ± 0.11 (*n* = 3)	0.44 ± 0.14 (*n* = 3)	0.27

WT, wildtype; KO, knockout. The results are expressed as the mean ± standard error of the mean. The right testis from each mouse was used.
